# The surgical symphony of human efficiency: a structured intraoperative workflow model for high-volume cochlear implant missions

**DOI:** 10.1016/j.bjorl.2026.101928

**Published:** 2026-10-14

**Authors:** Eman Hajr, Yassin Abdelsamad, Fida Almuhawas, Farid Alzhrani, Abdulrahman Hagr

**Affiliations:** a Imam Mohammad Ibn Saud Islamic University (IMSIU), Riyadh, Saudi Arabia; b MED-EL GmbH, Research Department, Riyadh, Saudi Arabia; c King Abdullah Ear Specialist Center (KAESC), King Saud University Medical City, King Saud University, Riyadh, Saudi Arabia

**Keywords:** Operating room, Cochlear implant, Hearing loss, Humanitarian, Green cochlea

## Abstract

**Objective:**

High-volume humanitarian Cochlear Implant (CI) missions require highly organized workflows to maintain efficiency and safety. This study describes the intraoperative model used during a large-scale CI campaign.

**Methods:**

A descriptive observational design was used to evaluate operative workflow across multiple operating rooms using standardized surgical protocols, structured shift-based assignments, and digital coordination. Operative times, intraoperative findings, ECAP responses, and postoperative radiologic outcomes were analyzed.

**Results:**

A total of 142 CI surgeries were completed over three days (30-, 66-, and 46-cases). The mean operative time was 57-minutes, with a median of 46-minutes. Unremarkable intraoperative fields were observed in 57.4% of cases, while perilymph oozing occurred in 31.2%. Absent ECAP responses occurred in three patients (2.1%) with inner ear malformations; all demonstrated responses during postoperative fitting. Radiologic assessment confirmed proper intracochlear insertion in all candidates.

**Conclusion:**

A structured intraoperative workflow enabled the safe and efficient completion of a high surgical volume within a short timeframe. This experience offers practical guidance for centers aiming to enhance operating room efficiency, particularly in high-volume or resource-limited environments.

**Level of Evidence:**

IV.

## Highlights

• High-volume CI mission: 142 cases completed in 3 days.
• Workflow model optimizes OR efficiency in CI surgery.
• Parallel operating rooms enable safe high-throughput surgery.
• Standardization reduces variability and delays in OR.
• Scalable model for humanitarian cochlear implant missions.

## Introduction

Operating Rooms (ORs) are among the most critical components of healthcare systems, with substantial influence on overall institutional performance and resource utilization. Efficiency in the operating room is a foundational element for any health system aiming to optimize outcomes and minimize costs. Achieving such efficiency requires a multidisciplinary approach with shared goals and mutual respect among all team members, including surgeons, anesthesiologists, circulating nurses, and scrub nurses ‒ who collectively contribute to maintaining a smooth intraoperative workflow.^1^^,^^2^

Considering that the OR is one of the most expensive areas of any hospital, efficient time management offers significant financial advantages by improving throughput and reducing costs per surgical encounter.^3^ This is especially relevant in low-resource environments, where limitations in staffing, infrastructure, and equipment magnify the consequences of inefficient OR scheduling or workflow interruptions.^4^

Humanitarian missions introduce an additional layer of complexity to OR management. In such settings, surgical teams operate within compressed timeframes and must be highly organized to achieve the targeted number of patients. Patients themselves often travel long distances, sometimes crossing international borders, and may have limited permissible durations of stay due to logistical, financial, or political constraints. These factors make efficient OR performance not only desirable but essential to mission success.

Humanitarian Cochlear Implant Missions (HCIMs) offer a valuable context for analyzing surgical workflow and time management strategies aimed at improving operative efficiency.

Cochlear Implants (CIs) are advanced electronic devices that restore hearing in individuals with severe to profound hearing loss and significantly enhance quality of life.^5^, ^6^, ^7^ However, military conflict and population displacement frequently disrupt healthcare systems and limit access to otologic, audiologic, and rehabilitative services, preventing children from receiving CI at the recommended early age.^8^^,^^9^ Such delays have long-term negative impacts on communication, education, and overall well-being. Similar challenges have been described in other humanitarian crises where national CI programs were interrupted or entirely absent.^10^

When planning HCIMs, numerous factors must be considered, and one of the most critical determinants of success is comprehensive pre-mission planning. Previous work has focused on establishing general guidelines for organizing humanitarian cochlear implant missions, including an eight-step framework that addresses funding, preparation, patient recruitment, and post-operative follow-up.^11^ However, these guides do not address intraoperative workflow or operating room management, which represent the core determinants of surgical throughput and mission success.

This study aims to describe a structured intraoperative workflow model designed to optimize efficiency and ensure safe and continuous surgical flow during a large-scale HCIM.

## Methods

### Study design

This study adopts a descriptive observational design to examine the intraoperative workflow and organizational structure of a large-scale Humanitarian Cochlear Implant Mission (HCIM). The design aims to document how surgical processes were arranged, coordinated, and executed in a real-world humanitarian context without introducing experimental interventions or comparison groups. All findings reflect the actual sequence of events, providing an accurate representation of surgical flow, case timing, and operational efficiency.

### Pre-mission site assessment

Prior to the mission, a dedicated advance team conducted a comprehensive site assessment to evaluate the suitability of the hosting surgical centers for high-volume cochlear implantation. This visit included an evaluation of the operating room layout, confirmation of equipment functionality, and an assessment of the facility’s capacity to support simultaneous multi-room activity. The team also identified which surgical instruments, disposables, and implant-related tools were available on site and which items needed to be supplied by the mission. Findings from this assessment informed the operational plan, OR allocation, and overall preparation for safe and efficient mission execution. After all, comes the decision for the best hospital among the different options that suet the mission more than the others.

### Instruments and tools

This hospital which was selected gets augmented by external support to have it ready before the mission. Surgical tools were minimized to crucial need to fasten the sterilization without compromising the needs. The final list of instruments was reviewed by all members of the team to approve them and approve the standard technique. Simulations were done as a team, so every member of the team knows his duty very clearly.

Medical instruments require custom clearance in many countries to get in and out the boarders very easily and fast. Some customs do not clear things in the weekend so early transportation to the country is required. Some customs require official letters including all instruments and duration of mission to get exemption.

### Team selection and eligibility

A multidisciplinary surgical team was assembled specifically for the mission, with each member selected based on predefined competency and experience criteria. All participating surgeons were fellowship-trained in otology and cochlear implantation, with substantial experience in pediatric CI surgery and high-volume operative settings. Anesthesia providers, scrub nurses, circulating nurses, and biomedical engineers were chosen for their technical expertise, familiarity with otologic procedures, and proven ability to function efficiently as a team within accelerated surgical workflows. This approach ensured that each operating room was staffed by individuals capable of delivering consistent performance while adhering to a unified surgical protocol and maintaining high patient-safety standards.

### Standardized preoperative protocol

Each patient was identified and confirmed in the holding area by both the surgeon and the anesthetists, including verification of name, procedure, and laterality. The surgical site was marked, and informed consent was reconfirmed to ensure full understanding of risks and benefits.

Patients were transferred to the operating room and positioned supine. A standard surgical safety checklist was completed. General anesthesia was induced via intravenous access and mask ventilation, followed by endotracheal intubation. Facial nerve monitoring electrodes were applied and tested preoperatively. All patients received a single intraoperative dose of antibiotics and steroids.

### Standardized surgical protocol

After sterile preparation and draping, the ear was taped forward. A minimally invasive postauricular approach, as described in our previous work was used for all candidates.^12^^,^^13^ Electrode type was selected preoperatively for each patient. Array length was determined using OTOPLAN-based measurement of cochlear duct length from preoperative imaging. Electrode design (Form-series vs. Flex-series) was selected according to the anticipated risk of intraoperative perilymph oozing or gusher, with Form-series electrodes preferentially used in cases at higher anticipated risk of these findings. A 3-cm postauricular incision was made, and hemostasis achieved using coagulation cautery. A Cat’s Paw retractor was used to elevate the skin flap, with dissection carried out using a Freer elevator until the temporalis fascia was identified. A cortical mastoidectomy was performed, identifying key landmarks including the lateral semicircular canal and short process of the incus. The implant’s silicone template was used to confirm exposure, and the receiver–stimulator bed was drilled to ensure a precise fit without fixation sutures.

The facial recess was opened using a diamond bur under microscopic guidance, with careful skeletonization of the facial nerve. Electrode insertion was performed through the round-window membrane and sealed with fascia. Closure was performed in layers using 3-0 Vicryl for the Palva flap and subcutaneous tissues, and 4-0 absorbable monofilament sutures for subcuticular skin closure. Opsite spray was applied.

Following closure, electrically Evoked Compound Action Potentials (ECAP) and impedance measurements were obtained and documented. All patients were extubated smoothly and transferred to recovery in stable condition.

### Standardized postoperative protocol

To streamline documentation, a standardized intraoperative notes template was used for all cases. The template included fixed fields for operative laterality, implant type, electrode measurements, insertion details, and intraoperative findings. Surgeons documented only variable data, ensuring accuracy while minimizing documentation time.

A standardized postoperative management form was also used for all patients, including medications, wound-care instructions, activation planning, and additional recommendations. This ensured consistent and complete discharge information for every patient.

### Workflow organization with defined roles (bee comb model)

A structured workflow model was established to maximize OR efficiency across all mission days. This model, referred to as the ‘Bee Comb Model,’ is based on parallel multi-room activity coordinated through centralized oversight and synchronized task allocation to maximize efficiency and minimize downtime.

On Day 1, OR activation was staggered due to concurrent patient evaluation, readiness confirmation, and completion of surgical consent. Consequently, surgeons followed a flexible rotation model, moving between rooms according to OR readiness and patient preparation. This dynamic approach allowed early cases to proceed without delay and accommodated the variable starting times inherent to the first mission day.

On Days 2 and 3, the workflow shifted to a high-efficiency operational model comprising two fixed shifts per day ‒ a morning and an evening shift ‒ each operating four fully staffed ORs. Surgeons were assigned to fixed rooms within each shift, supported by dedicated anesthesia teams, scrub nurses, circulating nurses, and biomedical engineers. This stable assignment minimized turnover delays and provided predictable workflow continuity.

### Parallel processing

To optimize efficiency, parallel processing was implemented. As the surgeon began wound closure, the anesthesia team proceeded to the preoperative area to assess and prepare the next patient. Conversely, when emergence from anesthesia began, the surgeon moved to evaluate the upcoming patient and confirm operative details. This bidirectional overlap minimized downtime and maintained steady case progression throughout all ORs.

### Leadership and oversight

A mission-wide OR supervisor provided continuous oversight across all operating rooms. Responsibilities included monitoring patient flow, verifying readiness of upcoming cases, ensuring instrument availability, and providing immediate assistance to any room experiencing delays. Patients were prepared in a dedicated preoperative holding area, and each OR was promptly cleaned and re-draped between cases. This coordinated system ensured seamless turnover and sustained high surgical throughput.

### Minimizing interruptions through digital coordination

To maintain an uninterrupted workflow, the mission employed a structured system of digital coordination. Surgeons, anesthesiologists, nurses, and biomedical staff communicated through secure digital channels, enabling real-time updates regarding patient readiness, instrument needs, and OR turnover without entering the operating room or interrupting ongoing procedures. This approach eliminated avoidable disruptions, reduced repeated inquiries, and ensured synchronized activity across the surgical team, ultimately supporting a continuous and efficient operative flow.

## Results

Across the three operative days, a total of 142 cochlear implant surgeries were completed: 30 cases on Day 1, 66 cases on Day 2, and 46 cases on Day 3.

### Patient characteristics

The cohort included 142 patients, with a male-to-female ratio of 57.7% (n = 82) to 42.3% (n = 60). The mean age was 4.0-years (range: 1–11 years), with a median age of 4-years. Most implants were performed on the right side (83.8%, n = 119), compared to 16.2% (n = 23) on the left (Table 1).

**Table 1:** Baseline demographic characteristics of implanted patients (n = 142).

Characteristic	Value
Total patients	142
Gender	82 males (57.7%)
	60 females (42.3%)
Mean age (years)	4.0
Median age (years)	4
Age range (years)	1–11
Implanted side	Right: 119 (83.8%)
	Left: 23 (16.2%)

### Inner ear anatomy

Most patients demonstrated normal cochlear anatomy (44.4%, n = 63), while 55.6% (n = 79) presented with inner ear malformations. The most common anomalies were Mondini deformity (26.8%, n = 38) and Incomplete Partition type II (IP-2) (24.6%, n = 35). Less frequent findings included cochlear hypoplasia (2.1%, n = 3), cochlear ossification (1.4%, n = 2), and common cavity deformity (0.7%, n = 1). The distribution of cochlear anatomy is illustrated in Fig. 1. All anatomical variations were successfully managed intraoperatively without deviation from the overall workflow.

**Fig. 1:**
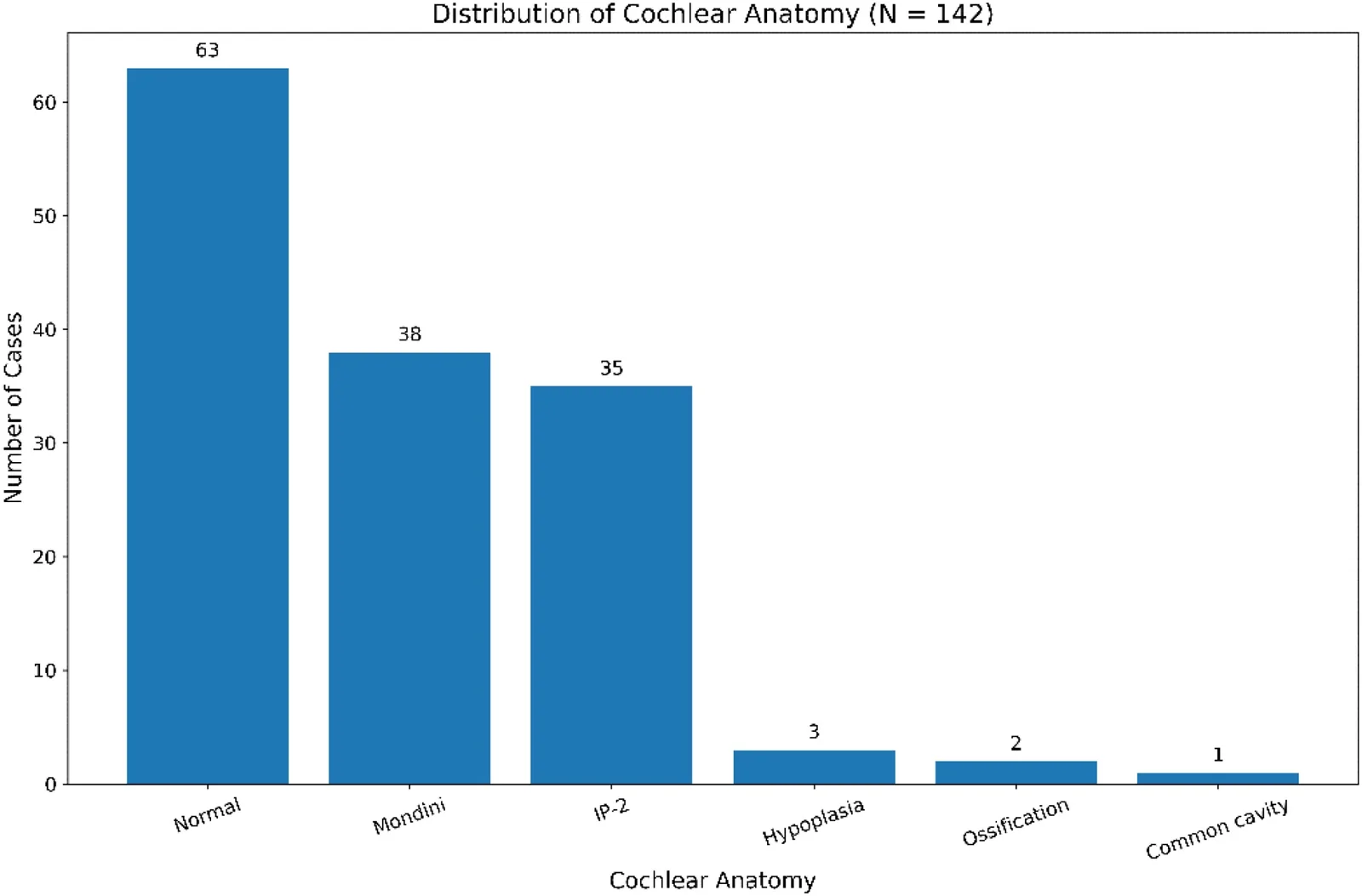
Distribution of cochlear anatomy among implanted patients (n = 142). The majority of patients demonstrated normal inner ear anatomy (44.4%), while inner ear malformations were identified in 55.6% of cases. The most common anomalies were Mondini deformity (26.8%) and Incomplete Partition type II (IP-2) (24.6%), followed by less frequent findings including cochlear hypoplasia (2.1%), cochlear ossification (1.4%), and common cavity deformity (0.7%).

### Operative time

Operative time analysis included all cases with complete time-in/time-out documentation. Despite the high surgical volume and multi-shift workflow, operative duration remained efficient and consistent throughout all mission days. A detailed summary of day-specific operative timings is presented in Table 2. Across all valid entries, the mean operative time was 57-minutes, while the median was 46-minutes.

**Table 2:** Operative time analysis across the three operative days.

Day	Total Surgeries	Mean (min)	Median (min)	IQR (min)
Day 1	30	53.2	48.7	38.6–69.8
Day 2	66	58.6	43.2	27.8–66.6
Day 3	46	57.5	51.4	32.8–69.8
Overall	142	57.0	46.2	32.1–69.8

### Intraoperative surgical findings

Most procedures demonstrated unremarkable intraoperative fields, accounting for 57% of cases. Mild perilymph oozing was observed in 31% of cases. Less frequent intraoperative findings included granulation tissue, resistance during electrode insertion, intraoperative gusher, and External Auditory Canal (EAC) defects. The full distribution of intraoperative findings is illustrated in Fig. 2.

**Fig. 2:**
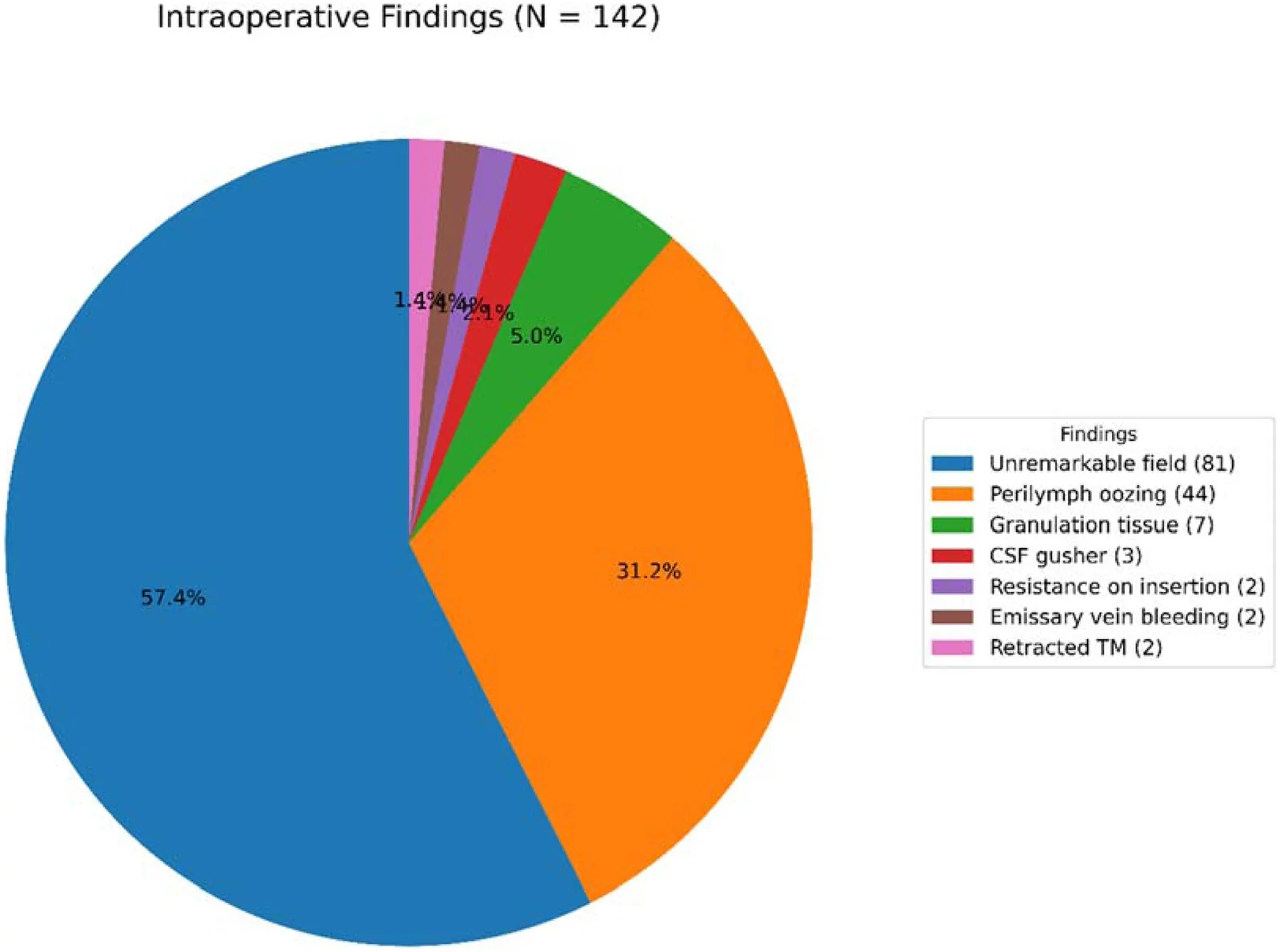
Distribution of intraoperative findings during cochlear implantation (n = 142). Most procedures demonstrated an unremarkable operative field (57.4%). Perilymph oozing was observed in 31.2% of cases. Less frequent intraoperative findings included granulation tissue (5.0%), Cerebrospinal Fluid (CSF) gusher (2.1%), resistance during electrode insertion (1.4%), emissary vein bleeding (1.4%), and retracted tympanic membrane requiring cartilage support (1.4%). Some patients exhibited more than one intraoperative finding.

### Intraoperative electrophysiological findings

Normal Intraoperative Measurement (ECAP/Impedance) was achieved in nearly all cases. However, three patients (2.1%) demonstrated absent intraoperative ECAP responses despite complete electrode insertion. All three had inner ear malformations, two cases of cochlear hypoplasia and one case of common cavity. In these three cases, ECAP responses were absent at all tested electrodes. Intraoperative radiography confirmed proper electrode position, and postoperative fitting (next day) revealed present ECAP responses in all three cases, indicating delayed but normal electrophysiologic detection. In six additional patients with incomplete intracochlear insertion (electrode 12 alone, or electrodes 11 and 12, located outside the cochlea on postoperative imaging), absent ECAP responses were limited to these extracochlear electrodes, while all remaining, fully inserted electrodes demonstrated normal responses. In all other patients, ECAP responses were present at all tested electrodes (100%), with no exceptions.

### Postoperative radiological assessment

Postoperative modified Stenver view radiographs confirmed appropriate electrode positioning in most patients:

• Full intracochlear insertion: 129 cases (90.8%).
• Radiologic over-insertion appearance: 4 cases (2.8%).
• Incomplete insertion, with distal electrodes 11–12 located outside the cochlea: 6 cases (4.2%).

All patients underwent anatomy-based fitting.

## Discussion

The present study builds upon the organizational principles outlined in the *Green Cochlea Guide*,^11^ advancing them by introducing, for the first time, a structured intraoperative workflow model specifically adapted for high-volume humanitarian cochlear implant missions. Although the mission was initially planned to extend over approximately five operative days, all surgeries were successfully completed within three days, underscoring the effectiveness of the organizational structure implemented.

Several key factors contributed to this accelerated yet safe workflow. First, the high competency of the surgical, anesthetic, nursing, and biomedical teams enabled consistent performance across all operating rooms. The appropriate allocation of specialized personnel, particularly anesthesiologists ‒ has been identified as a major determinant of operating room efficiency.^14^ Proper matching of the team members to specific surgical schedules is essential in minimizing turnover time, reducing delays, and preventing overutilization of OR resources. In the context of this mission, precise team assignment played a critical role in sustaining surgical flow and ensuring predictable room readiness, thereby enhancing overall operational performance.

Second, the adoption of a unified surgical protocol reduced inter-surgeon variability and allowed for predictable coordination across rooms. Third, the implementation of a shift-based operating model ensured sustained OR activity with minimal downtime, allowing anesthesia preparation, surgical evaluation, wound closure, and postoperative turnover to proceed in parallel. Together, these elements created a streamlined operative environment that enabled the mission to achieve high productivity without compromising safety.

Electrode selection in this cohort was determined preoperatively based on imaging-derived cochlear measurements and anticipated intraoperative findings, rather than adjusted in response to intraoperative events. Operative times were comparable among the electrode types used in anatomically normal or near-normal cochleae (Flex 26, Form 24, Flex 28). The Form 19 electrode, used in only five cases, was associated with a longer mean operative time; however, four of these five cases involved significant inner ear malformation (cochlear hypoplasia or common cavity deformity), for which this shorter array is preferentially selected. This difference in operative time therefore reflects the anatomic complexity of the underlying cases rather than a property of the electrode itself.

These findings align with the broader “Green Cochlea” concept, which highlights the importance of optimizing operating room practices to reduce waste, energy consumption, and overall environmental impact.^15^

Harders et al. (2006) demonstrated that redesigning operative processes can significantly reduce Non-Operative Time (NOT), with the greatest benefit observed in procedures lasting two hours or less.^16^ This aligns closely with our operational target of a 1-h skin-to-skin time, highlighting the importance of structured planning and optimized OR logistics in achieving efficient case progression.

Frequent interruptions are well-known contributors to prolonged operative time and reduced team performance. A study from Germany reported an average of nine interruptions per hour, with equipment failures and OR-environment disruptions posing the greatest interference with team functioning.^17^ Similarly, Weigl et al. (2015) found that repeated interruptions and irrelevant communication negatively affect the mental focus and cognitive load of surgical teams.^18^ In this mission, minimizing intraoperative interruptions was a deliberate objective. The use of a digital coordination system provided real-time updates on patient readiness, equipment needs, and OR status, thereby reducing the need for physical entry into the OR and avoiding unnecessary disruptions. Additionally, the availability of backup plans for equipment or instrumentation failure further protected workflow continuity, ensuring that no room experienced prolonged unplanned delays.

Finally, leadership dynamics play a recognized role in optimizing OR performance. Transformational leadership ‒ with its emphasis on open communication, teamwork, shared goals, and collaborative decision-making ‒ has been associated with improved surgical flow, reduced errors, and enhanced team cohesion.^19^ The centralized presence of an OR mission supervisor in this campaign enabled continuous oversight, rapid problem-solving, and seamless coordination across the four operative rooms, embodying the principles of effective perioperative leadership.

It is important to note that the current study emphasizes intraoperative workflow and operating room management, representing only one component of a broader, structured care pathway. All cases underwent comprehensive preoperative assessment and preparation prior to the mission. The methodology and outcomes of this preoperative phase, including the utilization of telemedicine, will be presented in separate publications.

Despite the strengths of this study and its demonstration of a highly organized and efficient intraoperative workflow, several limitations should be acknowledged. The mission was conducted by a carefully selected team, all of whom possessed substantial expertise in cochlear implant surgery and high-volume operative environments. This concentration of experience likely contributed to the mission’s success and may limit the generalizability of the findings to settings with fewer resources or less specialized personnel. Additionally, the operational model relied on the availability of multiple well-equipped operating rooms and a robust leadership structure ‒ factors that may vary across humanitarian programs and clinical centers.

Furthermore, this study does not extend to postoperative clinical outcomes, such as device activation responses, auditory performance, or long-term speech and language development. These outcomes are essential for evaluating the broader impact of high-throughput surgical models on patient quality of life and overall program effectiveness.

A comprehensive follow-up study is currently planned to assess both short- and long-term postoperative outcomes in this cohort.

## Conclusion

This study demonstrates that a structured intraoperative workflow can enable safe and efficient high-volume cochlear implant surgery in a humanitarian setting. The mission achieved 142 surgeries in three days with excellent intraoperative and radiologic outcomes.

Such an experience provides valuable guidance for any center aiming to improve operating room efficiency, particularly in high-volume environments.

Future work will focus on assessing short- and long-term postoperative outcomes to further evaluate the effectiveness and sustainability of this model.

## ORCID ID

Yassin Abdelsamad: 0000-0001-8281-5825

Fida Almuhawas: 0000-0002-3354-8756

Abdulrahman Hagr: 0000-0002-3561-6791

## Funding

This work did not receive specific research funding. The humanitarian mission was supported by the King Salman Humanitarian Aid and Relief Centre.

## Data availability

The authors declare that all data are available in repository.

## Declaration of competing interest

The authors declare that they have no known competing financial interests or personal relationships that could have appeared to influence the work reported in this paper.
